# Comparison of Hemodynamic Brain Responses Between Big Wave Surfers and Non-big Wave Surfers During Affective Image Presentation

**DOI:** 10.3389/fpsyg.2022.800275

**Published:** 2022-06-16

**Authors:** Mary Showstark, Ryan Bahadursingh, Sheng Zhang, Adam Fry, Barbara Kozminski, Per Lundstam, David Putrino

**Affiliations:** ^1^Yale School of Medicine Physician Assistant Online Program, New Haven, CT, United States; ^2^Red Bull North America, Santa Monica, CA, United States; ^3^Department of Psychiatry, Yale University School of Medicine, New Haven, CT, United States; ^4^Department of Rehabilitation and Human Performance, Icahn School of Medicine at Mount Sinai, New York, NY, United States; ^5^Department of Physical Medicine and Rehabilitation, University of Washington, Seattle, WA, United States

**Keywords:** fMRI, surfing, psychophysiological interaction, threat, fear

## Abstract

**Background:**

Big wave surfers are extreme sports athletes who expose themselves to life-threatening risk when training and competing. Little is known about how and why extreme sports athletes choose to participate in their chosen sports. This exploratory study investigated potential neurophysiological and psychometric differences between big and non-big wave surfers.

**Methods:**

Thirteen big wave surfers (BWS) and 10 non-big wave surfers (CON) viewed a series of images from the International Affective Picture System (IAPS) while undergoing brain functional magnetic resonance imaging (fMRI). The Fear Schedule Survey-III, Arnett Inventory of Sensation Seeking, Discrete Emotions Questionnaire, and Positive and Negative Affect Schedule were also completed.

**Results:**

The BWS group demonstrated higher blood-oxygen level-dependent (BOLD) signal change in the insula, visual cortex, and periaqueductal gray, whereas the CON group displayed increased hypothalamus activation in response to high amplitude negative-valence (HAN) image presentation. Psychophysiological interaction (PPI) analyses found CON showed significant interactions between frontal and temporal cortical regions as well as between the hypothalamus and the insula, frontal, and temporal cortices during HAN image presentation that were not seen in BWS. No differences between groups were found in their responses to the questionnaires.

**Conclusion:**

Our findings demonstrate significant differences in brain activation between BWS and CON in response to the presentation of HAN IAPS images, despite no significant differences in scores on psychometric questionnaires.

## Introduction

Extreme sports are activities that present a high level of risk to those participating (Willig, 2008). Elite extreme sports athletes comprise only a small subset of all elite athletes and are relatively understudied. As such, very little is known about how and why extreme sports athletes choose to participate in their chosen sports; theories range from sensation seeking and behavioral dependence to simple intrinsic motivation for skill mastery as potential motivations for extreme sports participation (Celsi et al., 1993; Bennett and Kremer, 2002; Partington et al., 2009; Wiersma, 2014). Fewer studies still have focused on brain hemodynamics in extreme sports athletes, how they respond to exposure to affective stimuli, and under what circumstances they may differ from a more normative population.

Individual responses to affective stimuli are known to be variable across individuals and context (Silvers et al., 2012). Previous studies have used functional magnetic resonance imaging (fMRI) to identify brain regions that may be involved in responding to affective image sets such as the International Affective Picture System (IAPS; Lang et al., 1997) or emotive faces (Ekman and Friesen, 1976). Exposure to affective stimuli produce hemodynamic responses in many brain regions including limbic and subcortical regions of the brain such as the amygdala (Breiter et al., 1996; Morris et al., 1996; Phillips et al., 1997; Liberzon et al., 2003), hypothalamus (Gunnar and Hostinar, 2015), periaqueductal gray (Mobbs et al., 2007), and insula (Paulus et al., 2010; Thom et al., 2012). In addition to lower brain areas, hemodynamic responses have also been observed in frontal cortical regions such as the anterior cingulate cortex (Killgore and Yurgelun-Todd, 2004; Britton et al., 2006), medial prefrontal cortex (Kim et al., 2003; Phan et al., 2003; Winston et al., 2003; Britton et al., 2006), ventromedial prefrontal cortex (Phan et al., 2004), and orbitofrontal cortex during exposure to affective stimuli (Blair et al., 1999). As such, it is unlikely that any single brain region is responsible for the processing of affective images. In fact, more recent literature suggests that the neural mechanisms associated with the processing of affective images involves activations of networks between multiple brain regions (Lindquist et al., 2012).

Few studies have investigated how patterns of hemodynamic responses to affective image presentation might differentiate elite performers from the normative population. Previous work involving elite athletes and military operators has highlighted some differences in hemodynamic responses to affective image exposure when compared with controls. For instance, U.S. Navy SEALs exposed to emotive faces showed increased insula activation overall and in response to angry faces as compared to non-SEAL controls (Paulus et al., 2010). Conversely, fMRI imaging of American college football players exposed to sports specific and negatively valanced images showed lower brain activity in the prefrontal cortex and insula compared to non-athlete controls (Costanzo and Hatfield, 2013). Finally, elite adventure racers showed increased activation in the right insula, left amygdala, and dorsal anterior cingulate cortex and decreased activation in the right medial prefrontal cortex in response to an emotional face processing task when compared to controls (Thom et al., 2012).

Further exploration of the neuropsychological processes of extreme sports athletes in response to affective stimuli may inform coaching strategies and performance psychology. Big wave surfing is an example of an extreme sport in which elite performers expose themselves to a significant risk of serious or fatal injury every time they participate. Extreme sport athletes within surfing may be of particular scientific interest as other highly proficient surfers who choose not to assume the risks of big wave surfing offer a seemingly well-matched control group, which is not the case for most other extreme sports (e.g., skydiving). The goal of this exploratory study was to compare and contrast both regional and networked hemodynamic brain responses to affective image presentation in big wave surfers in comparison with non-big wave surfers.

## Materials and Methods

### Participants

A convenience sample of 25 surfers were recruited for this study. Surfers were categorized into two groups: big wave surfers (BWS, *n* = 15) and non-big wave surfers (CON, *n* = 10). The BWS group included those who regularly surfed waves >20 feet in height (using the Hawaiian measurement system), whereas the CON group included those who had not surfed waves of this height. Of note, the Hawaiian measurement system measures the back of a wave, whereas other measurement systems, including that used by the National Weather Service, report wave heights by measuring the face of a wave. The face height of a wave may be approximately double the height of the back of the wave. Therefore, the 20-foot value used in our study to categorize surfers as big wave surfers would correspond to a substantially higher value if referring to the face of the wave. Our study used the Hawaiian method of measurement as it was more commonly used and easily comprehended within our target population.

Of the 25 surfers recruited for this study, one participant was withdrawn due to experiencing claustrophobia upon entering the fMRI scanner and another was excluded because they fell asleep during the fMRI scan. Subsequently, 23 participants were included in the analyses (Table 1).

**Table 1:** Participant characteristics.

	BWS	CON
*n*	13	10
Age (years)	32 ± 9	35 ± 7
Male/female	10/3	9/1
Professional or semi-professional (n)	12	1^*^
Surfing experience (years)	26 ± 8	21 ± 10
Current surfing activity (hours/week)	13 ± 10	6 ± 3^**^

*Values are mean ± standard deviation*.

* *p < 0.001, Chi-squared test*.

** *p < 0.05, Independent samples *t*-test*.

All study procedures were completed at the Invision Imaging Center in Honolulu, Hawaii. All participants provided written informed consent prior to participation, and the experimental protocol was fully approved by the institutional review board of Yale University (IRB #2000024944).

### Behavior Task

Participants viewed a series of images from the International Affective Picture System (IAPS) while undergoing brain fMRI. The IAPS is a standardized, emotionally evocative photo set specifically designed for experimental neuropsychology studies (Lang et al., 1997). IAPS photos carry ratings based on various factors including valence and arousal, meaning comparisons of responses to positive or negative affect can be investigated separately. IAPS images were categorized according to valence (positive or negative) and arousal ratings (high or low), creating four groups: high arousal positive (HAP), high arousal negative (HAN), low arousal positive (LAP), and low arousal negative (LAN). Forty images were selected from each of these groups for a total of 160 images (Appendix). Participants were asked to view all images presented on the screen. No tasks related to regulation were instructed. No real-time feedback (e.g., subjective valence or arousal ratings) or behavioral responses (e.g., button press) were performed. After the fMRI, participants were asked whether they were able to remain attentive to all images with anyone who fell asleep for any duration excluded from the analysis.

Participants viewed the images using a pair of MRI compatible goggles (CinemaVision, Resonance Technology Inc., Northridge, CA). To account for a color saturation effect when viewing the images on the goggles, all photographs were first edited to reduce saturation by 30%. Images were presented in a randomized order using an in-house script in PsychoPy3 (Peirce et al., 2019). We used an event-related design with jittered inter-stimulus interval in current study. In modeling, the event (image presentation) onset was convoluted with the hemodynamic response function to capture image-related brain responses. Each image presentation trial was 2.5 s in length. The time at which an image appeared within each trial was determined using a randomized jitter of 0.1, 0.3, or 0.5 s. The image was then displayed for 1 s. A light gray fixation cross was displayed against a black background while images were not displayed. An additional 20 trials were included in which no image was presented, and participants continued to view the fixation cross (FIX trials). The order of the FIX trials among the image trials was also randomized. Thus, the behavioral task consisted of 180 trials, lasting 7.5 min.

### MRI Data Acquisition

Imaging was performed using a 3 T MRI scanner (Achieva, Philips, Best, Netherlands). Participants were screened for metal or other surgical implants prior to entering the MRI room. A 7-min anatomical MRI scan was completed in addition to the fMRI. Anatomical images of the functional slice locations were obtained with gradient echo sequences (GRE) in the sagittal plane with TR = 8.1 ms, TE = 3.7 ms, bandwidth = 191.4 Hz/pixel, flip angle = 8°, field of view = 240 × 240 mm, matrix = 240 × 240, 145 slices with slice thickness = 1 mm and no gap. Functional, blood-oxygen level-dependent (BOLD) signals were acquired with a multi-shot gradient echo echoplanar imaging (EPI) sequence. Twenty-two axial slices covering the whole brain were acquired with TR = 2000 ms, TE = 30 ms, bandwidth = 56.6 Hz/pixel, flip angle = 75°, field of view = 240 × 240 mm, matrix = 64 × 62, 22 slices with slice thickness = 5 mm and no gap.

### Imaging Data Preprocessing

Data were analyzed using Statistical Parametric Mapping (SPM12). Images of each individual subject were first realigned (motion corrected) and corrected for slice timing. A mean functional image volume was constructed for each subject per run from the realigned image volumes. Subjects whose head motion exceeded 3.0 mm in translation or 3 degrees in rotation were excluded. These mean images were co-registered with the high-resolution structural image and then segmented for normalization with affine registration followed by nonlinear transformation. The normalization parameters determined for the structure volume were then applied to the corresponding functional image volumes for each subject. Finally, the images were smoothed with a Gaussian kernel of 8 mm at full width at half maximum.

### Imaging Data Modeling

We distinguished five trial conditions: HAN, HAP, LAN, LAP, and FIX. A statistical model was constructed for each individual subject using a general linear model (GLM) with image onset in each trial convolved with a canonical hemodynamic response function and with its temporal derivative for entry as regressors in the model (Friston et al., 1995). Realignment parameters in all six dimensions were entered in the model. The data were high-pass filtered (1/128 Hz cutoff) to remove low-frequency signal drifts. Serial autocorrelation was corrected by a first-degree autoregressive or AR (1) model. The GLM estimated the component of variance that could be explained by each of the regressors. In the first-level analysis, we constructed for each individual subject statistical contrasts of “HAP vs. FIX,” “HAN vs. FIX,” “LAP vs. FIX,” “LAN vs. FIX,” “HAP vs. LAP,” and “HAN vs. LAN.” These contrasts allowed us to evaluate brain regions that responded differently to viewing of affective images, as compared to fixation cross.

### Psychophysiological Interactions

Psychophysiological interaction (PPI) describes functional connectivity between brain regions contingent on a psychological context (Friston et al., 1997; Gitelman et al., 2003). We used a generalized form of context-dependent PPI (McLaren et al., 2012). Briefly, in generalized PPI, the hemodynamic responses of HAP, HAN, LAP, LAN, and FIX formed the psychological regressors, whereas in standard PPI, only contrasts (e.g., HAP > FIX) are included in the GLM. The inclusion of task regressors in generalized PPI reduces the likelihood that the functional connectivity estimates were driven by simple co-activation. The extracted mean time series of the BOLD signal were temporally filtered, mean corrected, and de-convolved to generate the time series of the neural signal for a mask for each individual subject to compose the physiological variable. These time series of neural signal were then multiplied by the onset times of the HAP, HAN, LAP, LAN, and FIX separately and re-convolved with the canonical hemodynamic response function to obtain the interaction term or PPI variable (Gitelman et al., 2003). Finally, brain regions showed significant differences between BWS and CON were used as seed regions for PPI analysis (see Results). The psychological regressors, the physiological variable of the region of interest, and PPI variables of HAP, HAN, LAP, LAN, and FIX were entered as regressors in a whole-brain GLM. Generalized PPI analysis was performed for each individual subject, and the resulting positive contrast images were used in random-effect group analysis (Penny et al., 2004).

### Group Analysis

For the group-level BOLD analyses, the contrast images from the individual-level analyses (above) were used. We conducted 2 (group: BWS vs. CON) by 2 (arousal: high vs. low) by 2 (valence: negative vs. positive) ANOVA model to examine the main effects of group, arousal, and valence as well as the interaction effect. In post-hoc analysis, we conducted one-sample t-tests across all 23 surfers and two-sample t-tests between BWS and CON to examine whole-brain activations related to contrasts “HAP vs. FIX,” “HAN vs. FIX,” “LAP vs. FIX,” “LAN vs. FIX,” “HAP vs. LAP,” and “HAN vs. LAN.” Further, we conducted two-sample t-tests between BWS and CON to examine altered functional connectivity for those brain regions showing significant differences of BWS vs. CON. Viewing signal change in the hypothalamus and periaqueductal gray was of interest due to existing literature highlighting their roles in processing negative-valence images (Gunnar and Hostinar, 2015; Lefler et al., 2020). However, because of the small size of these two brain regions, we specifically tested the statistics within a hypothalamus from the WFU Pick Atlas^1^ and a periaqueductal gray mask from the Harvard Ascending Arousal Network (AAN) Atlas (Edlow et al., 2012) using small volume correction.

### Questionnaires

Following the MRI, participants completed a series of questionnaires. The Discrete Emotions Questionnaire (DEQ) was used to measure participants’ subjective emotional responses to the IAPS images. The Arnett Inventory of Sensation Seeking (AISS) measured participants’ self-reported levels of sensation seeking across two scales: intensity of an experience and novelty of an experience. The Positive and Negative Affect Schedule (PANAS) assessed participants’ positive and negative feelings experienced over the past week. Finally, the Fear Schedule Survey-III (FSS-III) was used to assess self-reported levels of fear toward various circumstances, objects, or other stimuli. The questionnaire responses of the BWS and CON groups were compared using independent samples t-tests.

## Results

### Patterns of Signal Change During Negative-Valence IAPS Image Presentation

**Figure 1:**
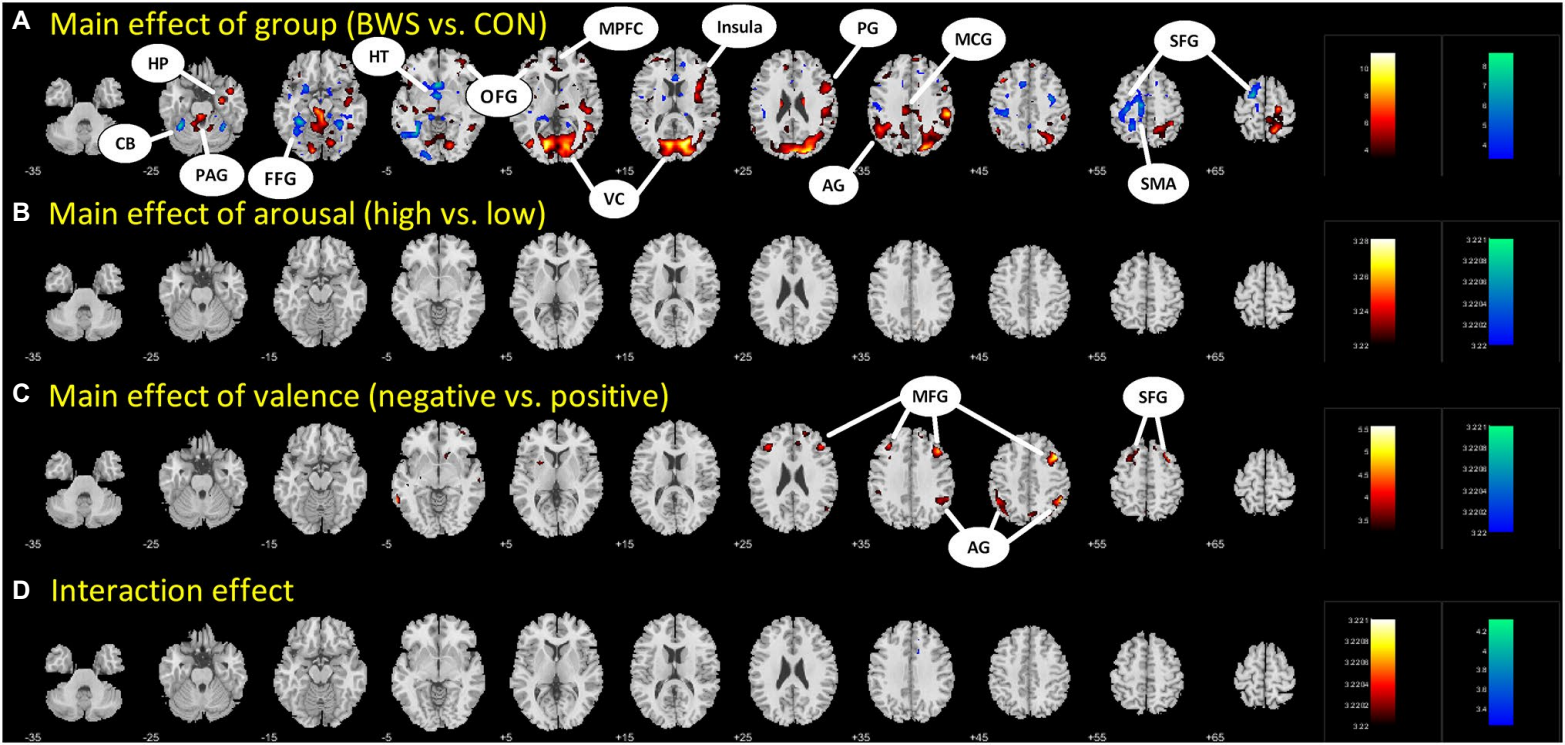
Difference in brain region activations between **(A)** BWS and CON participant groups across all image presentations, **(B)** arousal levels of the images (High vs. Low), **(C)** valence of the presented images (negative vs. Positive), and **(D)** interaction effects. CB, Cerebellum; HP, Hippocampus; PAG, Periaqueductal gray; FFG, Fusiform gyrus; HT, hypothalamus; OFG, Orbitofrontal gyrus; MPFC, Medial prefrontal cortex; VC, Visual cortex; PG, Precentral gyrus; AG, Angular gyrus; MCG, Middle cingulate gyrus; SFG, Superior frontal gyrus; SMA, Supplementary motor area; and MFG, Middle frontal gyrus.

Signal changes in response to affective image presentation were identified in localized regions of the brain at voxel *p* < 0.001 uncorrected and cluster-level *p* < 0.05, FWE corrected. When exploring differences in mean BOLD signal between BWS and CON groups, the BWS group showed significant increases in BOLD signal in the visual cortex, periaqueductal gray, hippocampus, frontal areas of cortex, insula, and compared to the CON group (Figure 1A). By contrast, the CON group showed significant increases in BOLD signal in the cerebellum, hypothalamus, and frontal motor regions compared with BWS group (Figure 1A). The arousal level of affective images did not result in any observable brain signal changes (Figure 1B). Exposure to positive valence images resulted in minimal brain signal changes, while presentation of negative-valence images resulted in significant increases in signal intensity across all participants in the middle frontal gyrus, angular gyrus, and superior frontal gyrus (Figure 1C). No significant interaction effect was observed (Figure 1D). All responses are reported in detail in Table 2.

**Table 2:** Regions showing activations in ANOVA model of group (BWS vs. CON) by arousal (high vs. low) by valence (negative vs. positive).

Volume(mm^3^)	Peak voxel(Z)	MNI coordinates (mm)			Side	Identified brain region
		*x*	*y*	*z*				
*Main effect of group: BWS > CON*						
122,850	10.13	−15	−76	7	L/R	Visual cortex, Vermis, Periaqueductal gray
5,805	6.90	30	−10	−29	R	Hippocampus
4,887	6.82	30	44	−8	R	Orbitofrontal gyrus
7,749	6.72	−48	−52	37	L	Angular gyrus
14,256	6.32	48	2	28	R	Precentral gyrus, Insula
8,802	5.71	6	53	37	L/R	Medial prefrontal gyrus
3,483	5.08	−15	−46	37	L/R	Middle cingulate gyrus
2,835	4.50	−33	29	−14	L	Orbitofrontal gyrus
*Main effect of group: CON > BWS*						
12,042	6.97	−36	−49	−26	L	Cerebellum
9,342	6.62	−6	−1	−8	L/R	Hypothalamus
21,141	6.55	−18	−1	64	L	Supplementary motor area, Superior frontal gyrus
5,427	6.06	30	−46	−17	R	Fusiform, cerebellum
*Main effect of arousal: high > low*						
None						
*Main effect of arousal: low > high*						
None						
*Main effect of valence: negative > positive*						
7,452	4.98	42	20	43	R	Middle frontal gyrus
5,616	4.70	51	−46	46	R	Angular gyrus
2,781	4.58	−33	32	31	L	Middle frontal gyrus
4,482	4.01	−45	−49	43	L	Angular gyrus
2,403	3.64	−21	5	52	L	Superior frontal gyrus
*Main effect of valence: positive > negative*						
None						
Interaction effect						
None						

*Voxel *p* < 0.001 and cluster-level *p* < 0.05 whole-brain corrected for family-wise error of multiple comparisons. R, right; and L, left*.

**Figure 2:**
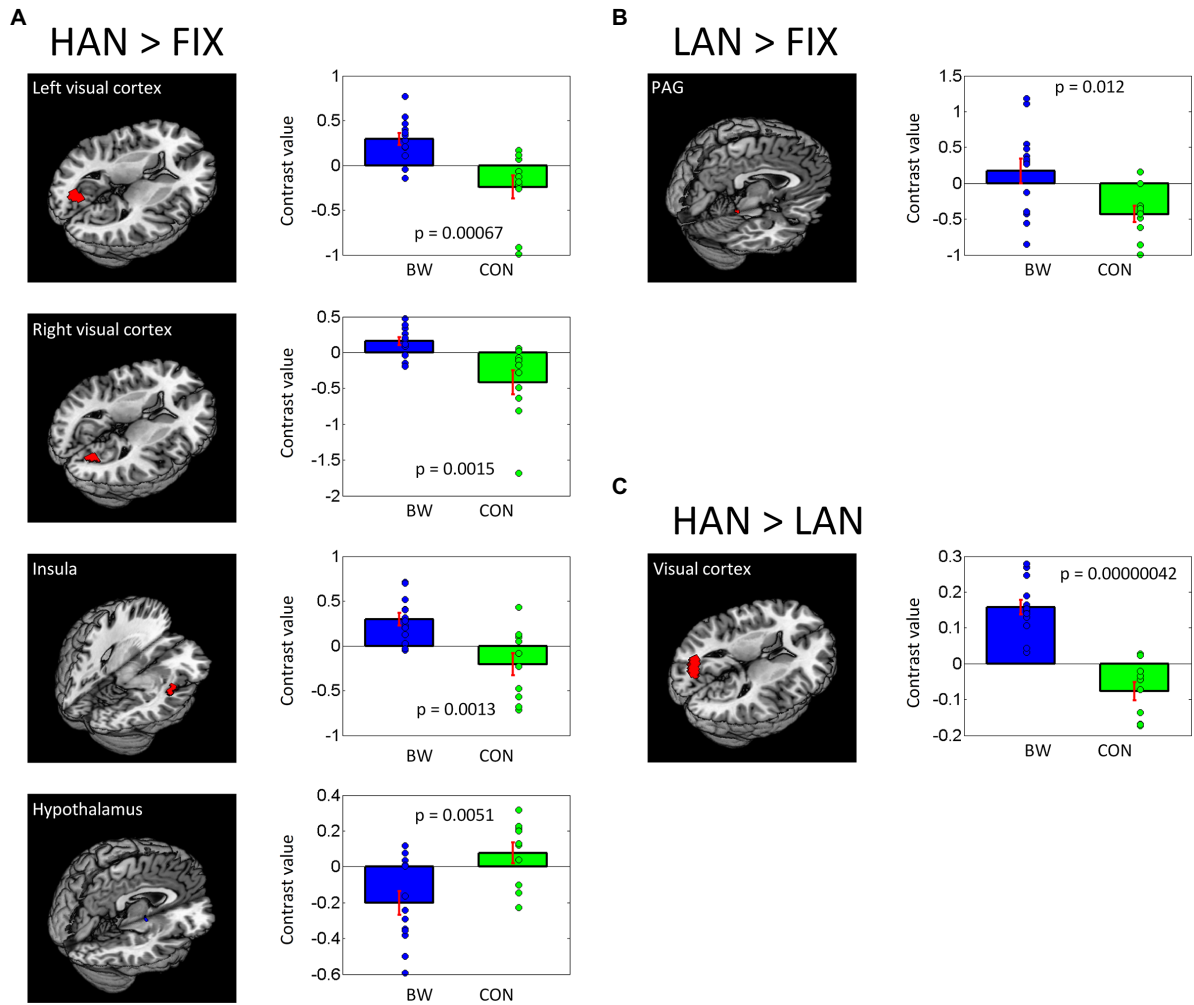
Differences in brain region activations between BWS and CON participant groups during negative IAPS image presentations **(A,B)**, as well as when responses to HAN and LAN conditions are compared **(C)**. Individual contrast values (blue and green diamonds) as well as average contrast values (blue and green bars) are shown.

In post-hoc analysis, significant differences in signal intensity were identified at voxel *p* < 0.005 and *p* < 0.05 AlphaSim correlation for multiple comparisons when BWS responses were compared to CON responses during HAN and LAN affective image presentation. Of note, significantly increased activation was observed in the visual cortex and the insula of the BWS group compared with CON during the presentation of HAN images (Figure 2A). During the presentation of HAN images, the CON group showed significantly increased activity in the hypothalamus when compared with responses from the BWS group at voxel p < 0.005 and p < 0.05 small volume correlation for the hypothalamus mask (Figure 2A). Finally, significantly increased activation of the periaqueductal gray was observed in the BWS group compared with the CON group during LAN image presentation at voxel p < 0.005 and p < 0.05 small volume correlation for the periaqueductal gray mask (Figure 2B). All responses are reported in detail in Table 3.

**Table 3:** Regions showing differences in activations between BWS and CON with age as a covariate.

Volume(mm^3^)	Peak voxel(Z)	MNI coordinates (mm)			Side	Identified brain region
		*x*	*y*	*z*		
*BWS > CON (HAN* vs. *FIX)*						
1,863	3.60	−18	−76	7	L	Visual cortex
1,053	3.44	45	−10	−11	R	Insula
1,350	3.32	18	−73	13	R	Visual cortex
*CON > BWS (HAN* vs. *FIX)*						
81^*^	3.22	6	−4	−8	L/R	Hypothalamus
*BWS > CON (LAN* vs. *FIX)*						
81^**^	2.65	3	−37	−17	L/R	Periaqueductal gray
*CON > BWS (LAN* vs. *FIX)*						
None						
*BWS > CON (HAN* vs. *LAN)*						
2,241	4.93	−18	−76	10	L	Visual cortex
*CON > BWS (FIX > HAN)*						
None						

*Voxel *p* < 0.005 and *p* < 0.05 AlphaSim correlation for multiple comparison*.

* *Voxel *p* < 0.005 and *p* < 0.05 small volume correlation for hypothalamus mask*.

** *Voxel *p* < 0.005 and *p* < 0.05 small volume correlation for periaqueductal gray mask; R, right; and L, left*.

### Differences in PPI Between BWS and CON During HANS Presentation

**Figure 3:**
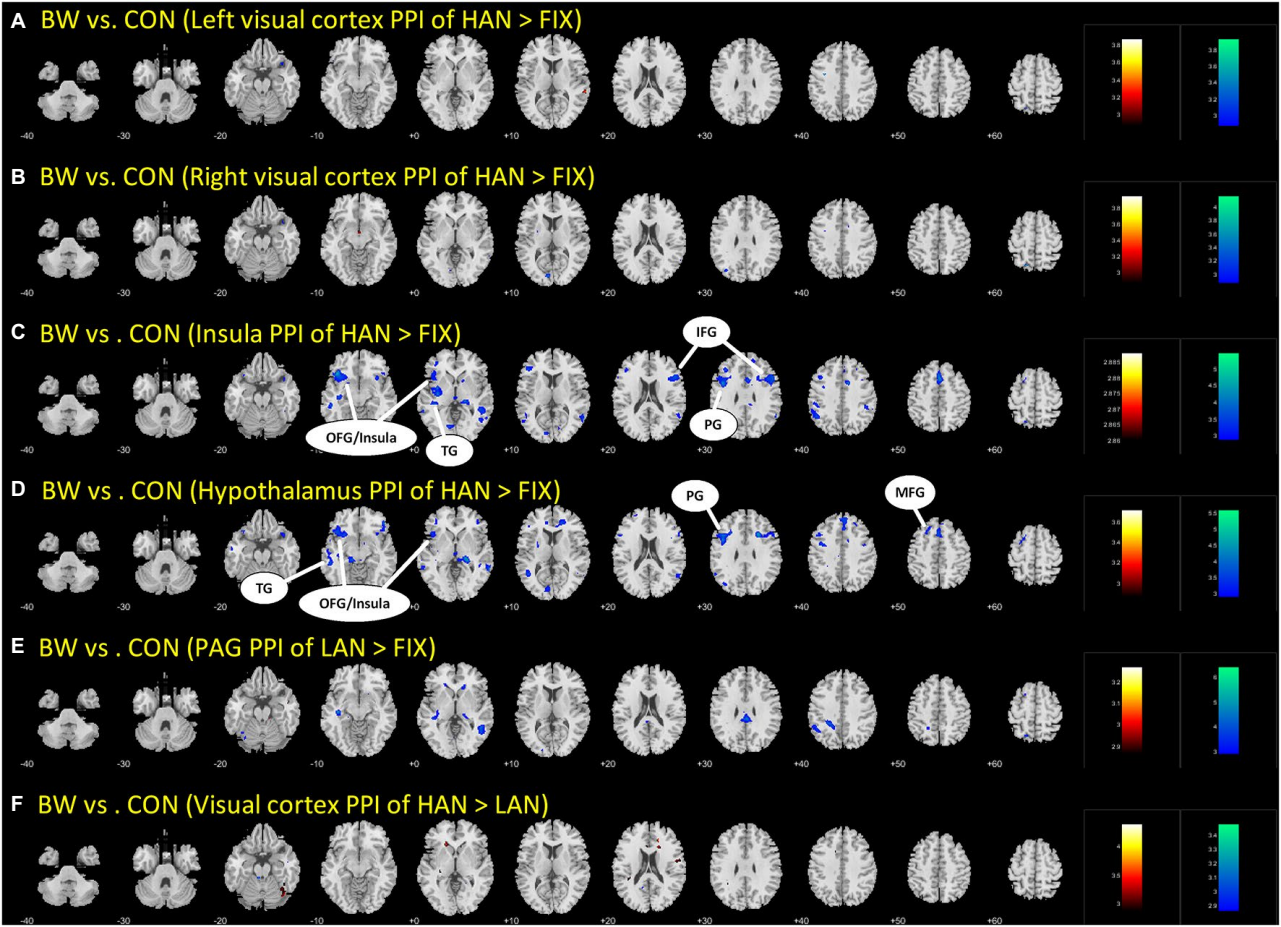
The different parts **(A-F)** are simply the different brain regions and conditions tested in the PPI analysis. Differences in PPI between BWS and CON during HAN image presentation. OFG, Orbitofrontal gyrus; TG, temporal gyrus; IFG, inferior frontal gyrus; PG, Precentral gyrus; and MFG, Middle frontal gyrus.

Brain regions showing differences between BWS and CON as in Table 3 were used as seed regions for PPI analysis. Patterns of significantly differing PPI between BWS and CON groups were observed at voxel *p* < 0.005 uncorrected and cluster-level *p* < 0.05. FWE corrected when the hypothalamus and insula were used as seed regions for the PPI. No difference was observed for other seeds. In both cases, the CON group showed significantly increased incidence of PPIs (Figure 3; Table 4). Notably, in the CON group, evidence of functional connectivity during HAN presentation was seen between the insula and the orbitofrontal, inferior frontal and superior temporal cortices, and the prefrontal gyrus. In addition, during HAN image presentation, the CON group also showed significantly increased PPI between the hypothalamus and the insula, superior temporal cortex, precentral gyrus, and middle frontal cortex.

**Table 4:** Regions showing differences in PPI between BWS and CON with age as a covariate.

Volume(mm^3^)	Peak voxel(Z)	MNI coordinates (mm)			Side	Identified brain region
		*x*	*y*	*z*		
**Insula PPI of HAN vs. FIX**						
*BWS > CON*						
None						
*CON > BWS*						
15,714	4.18	−39	26	−8	L	Orbitofrontal gyrus/Insula
	4.14	−36	35	28	L	Inferior frontal gyrus
	3.86	−51	−34	−5	L	Temporal gyrus
5,967	3.81	51	20	25	R	Inferior frontal gyrus
4,482	3.60	−42	8	31	L	Precentral gyrus
**Hypothalamus PPI of HAN vs. FIX**						
*BWS > CON*						
None						
*CON > BWS*						
4,914	3.78	−27	17	−14	L	Orbitofrontal gyrus/Insula
5,319	3.76	−42	8	31	L	Precentral gyrus
5,751	3.71	−24	20	52	L	Middle frontal gyrus
5,751	3.65	−54	−37	−5	L	Temporal gyrus

*P < 0.005 and cluster-level *p* < 0.05 whole-brain corrected for family-wise error of multiple comparisons; R, right; and L, left*.

### Questionnaire Responses

No significant differences between BWS and CON were observed for any of the questionnaire results. This included scores on the FSS-III (BWS: 126 ± 38; CON: 138 ± 27; t_21_ = 0.84, *p* = 0.41), AISS – novelty subscale (BWS: 30 ± 3; CON: 31 ± 4; t_21_ = 0.17, *p* = 0.87), AISS—intensity subscale (BWS: 31 ± 6; CON: 28 ± 3; t_21_ = 1.67, *p* = 0.11), PANAS—positive affect (BWS: 32 ± 6; CON: 31 ± 3; t_21_ = 0.28, *p* = 0.78), and PANAS—negative affect (BWS: 27 ± 5; CON: 26 ± 5; t_21_ = 0.86, *p* = 0.40). Results were also similar between groups for all emotions on the DEQ. Both groups reported relaxation to be the emotion they experienced to the greatest extent and fear to the least extent.

## Discussion

This exploratory study was the first to compare neurophysiological and psychometric measures between a cohort of BWS and CON participants. We identified significant differences in the fMRI-derived brain responses of BWS participants compared with CON participants during the presentation of negative affective images. These differences were observed in the absence of significant differences in psychometric evaluations between the two cohorts. It would appear that the major differences in fMRI results between the two groups were related primarily to areas of the brain that are concerned with threat response and regulation.

### Population Signal Change Responses to IAPS Images

Population responses to HAN images showed activity in various brain regions that are typically associated with threat processing and evaluation such as the striatum and supplementary motor areas for response planning and readiness (Butler et al., 2007; Almeida et al., 2013). Activation of the left angular gyrus is a less common response to HAN image presentation but has been previously reported during the presentation of threatening images, especially if those images trigger affective memory recollection (Fischer et al., 1996; Ramanan et al., 2018). However, it was not formally established whether our cohort had experienced traumatic experiences that may have been triggered by the IAPS image presentations. A notable lack of signal change in the amygdala was observed during HAN image presentation. Absences of amygdala activity has been described in individuals with generalized anxiety disorder (Prater et al., 2013) and individuals with higher levels of social inhibition (Blackford et al., 2014). Traditionally, presentation of HAN IAPS images is often accompanied by increased blood flow in the amygdala (Hariri et al., 2002). It is notable, therefore, that it is absent in this cohort. Results of the DEQ showed both groups reported fear to be the emotion they experienced to the least extent while viewing the IAPS images. In addition, although no significant difference was seen between athlete groups in FSS-III scores, both athlete groups scored significantly lower on the FSS-III than normative scores that have previously been reported (see: *responses to questionnaires*), indicating less generalized phobic behavior in comparison with the general population (Grossberg and Wilson, 1965). This paired with the observed lack of signal change in the amygdala may indicate that HAN images simply appear less threatening to this population than the general population. This assertion is hard to verify, though, since few other studies have captured the FSIII in conjunction with IAPS presentation.

### Signal Change Differences Between BWS and CON Participants

Significant signal change differences between the two groups of surfers in response to HAN images were identified, with the BWS cohort showing significantly increased signal change in the insular during HAN presentation when compared with CON. The insula has been identified as a key area in evaluating risk and making risky decisions. Others have shown that prior risk experience increases insula activity during risk evaluation, and the interaction between perceived threat and generating a bodily response to those threats (Wiech et al., 2010; Xue et al., 2010). Thus, increased activation of the insula in BWS compared with CON may indicate that BWS are more proficient in identifying, evaluating, and responding to risk and risky situations than their CON counterparts. Similarly, the BWS group showed greater activation of the medial prefrontal gyrus and the middle cingulate gyrus and orbitofrontal gyrus than the CON group. These areas have previously been shown to exhibit increases in hemodynamic brain responses during the assessment and regulation of threats (Eippert et al., 2007; Rubino et al., 2007; Fiddick, 2011). As such, increased activation of these brain regions observed in the BWS group may indicate they are more adept at evaluating and regulating threat than those in the CON group. In addition, BWS showed more visual cortex activation during HAN image presentation than CON. Strong visual cortex activation is common during visual threat presentation (Hofmann et al., 2012; Maratos et al., 2012; Miskovic and Keil, 2013), and significantly heightened visual cortex responses to visual threat presentation in the BWS are potentially indicative of a heightened awareness of threat (Hofmann et al., 2012). The CON group displayed increased signal change compared with the BWS group in the hypothalamus during presentation of the negative-valence images, while members of the BWS group displayed stronger activation in the periaqueductal gray. As a part of the hypothalamic–pituitary–adrenal axis, the hypothalamus is known to be involved in the planning of threat response (Mobbs et al., 2009; Gunnar and Hostinar, 2015) and response to psychosocial stress (Foley and Clemens, 2010). By contrast, the periaqueductal gray is more concerned with immediate survival responses to threat and enaction of escape behaviors (Mobbs et al., 2007, 2009; Lefler et al., 2020). The subtle difference in preferential activation of these brain regions may indicate a tendency for faster and more decisive response to threat in the BWS group. Similarly, the observation of increased signal change in the hypothalamic region of the CON group during negative affective image presentation may indicate that they were more strongly activating networks associated with planning escape behaviors, while the BWS group was concerned with enacting them. Taking the cortical and subcortical findings together, during the presentation of HAN images, BWS show stronger signal changes associated with forebrain regions involved in perceiving regulating and evaluating a threatening stimulus, while the CON group showed stronger activation in areas associated with planning escape strategy. These findings indicate significant differences in brain region activation between the two groups in response to the presentation of HAN images.

### Psychophysical Interactions During Affective Image Presentation

We observed significant PPI interactions between multiple brain regions during HAN image presentation in CON that were not present in BWS. Notably, in CON, the insula showed significant PPIs to frontal and temporal cortical regions, which has been identified previously in healthy adults regulating during aversive image presentation (Veit et al., 2012). The lack of PPIs in response to the same stimuli in BWS may indicate a difference in regulation strategies utilized by participants in the two groups. Similarly, the hypothalamus also showed significant PPIs between the insula, frontal, and temporal cortices during HAN image presentation in CON compared with BWS. Engagement of PPIs in the hypothalamus during negative affective image presentation is a novel finding that has not been previously reported in other studies of affective image presentation and fMRI responses. However, activation of these areas during threat and negative affective image presentation is not unprecedented. Taken together, these findings show that while participants in the CON group display evidence of PPIs that are commonly associated with emotion regulation in response to threat, BWS participants did not. Similar observations have been shown in elite and non-elite military as well as extreme sports athletes, with less-experienced performers displaying higher levels of physiological reactivity to threatening or stressful situations (Clemente-Suárez et al., 2017; Tornero-Aguilera et al., 2017). Of note, however, there was no significant difference in years of experience surfing between the BWS and CON cohorts, indicating that although the two groups had an equivalent level of experience, the respective difference in threat associated with participation in the sport between the two groups may be driving the neurophysiological differences seen.

### Responses to Questionnaires

Participant responses on the FSS-III did not differ between groups. However, participants in the present study scored lower on the FSS-III when compared to normative scores (156 ± 35; Grossberg and Wilson, 1965). Thus, while big wave and non-big wave surfers may experience fewer generalized phobias compared to the general population, number of phobias did not differ between surfers who did and did not surf big waves. AISS responses were also similar between groups. This is consistent with a previous study that found no differences in sensation seeking scores on the AISS among a sample of rock climbers who attempted climbs of different levels of difficulty (Aşçi and Demirhan, 2007). In addition, other research has indicated minimal differences in AISS scores between athletes who participate in high and low risk sports (Zarevski et al., 1998). However, AISS scores of both CON and BWS were higher than scores previously reported for a normative population (48 ± 8; Carretero-Dios and Salinas, 2008), indicating a higher level of sensation seeking in surfers compared to the general population. Though our fMRI results may suggest a greater proficiency among BWS to employ strategies for identifying, processing, and responding to threat, the lack of between-group differences exhibited in our questionnaire data may indicate that this process is unrelated to subjective ratings of the IAPS stimuli, experiences of phobias, or self-reported sensation seeking.

### Limitations

The present study had several limitations. The sample size was small, but this is common in studies involving elite performers due to the fact that elite performers are rare. For instance, our sample size of 13 big wave surfers likely represents 5–10% of the total, global BWS population, making the possibility of recruitment of a larger sample size challenging. Our CON group consisting of experienced non-big wave surfers displayed responses to affective HAN images and psychometric surveys that were similar to the BWS group, but represented deviations to what has been described previously in the literature in normative populations. This was an unexpected finding. Thus, a limitation of the present study was the lack of inclusion of a non-surf control group as a comparator. Finally, this was an exploratory study, meaning that much of the work was not strongly hypothesis-based. As such, we had a very small number of fixation trials relative to affective image trials due to the exploratory nature of the trial paired with the relatively large number of image types being evaluated. Thus, the increased activation in visual cortex during fixation vs. image trials could be due to the salience effect of the fixation images. Finally, many of the novel findings in this study related to hemodynamic responses in the hypothalamus and periaqueductal gray, which are small brain regions. Given the relatively low spatial resolution of these scans, it is possible that some of the differences observed between groups may be attributable to the partial volume effect. A replication of these exploratory findings is recommended using higher field MRI technology.

## Data Availability Statement

The raw data supporting the conclusions of this article will be made available by the authors, without undue reservation.

## Ethics Statement

The studies involving human participants were reviewed and approved by Institutional Review Board of Yale University. The patients/participants provided their written informed consent to participate in this study.

## Author Contributions

MS assisted with experimental design, data analysis and interpretation, and manuscript preparation. RB assisted with data collection, data analysis and interpretation, and manuscript preparation. SZ assisted with data analysis and interpretation and manuscript preparation. AF assisted with experimental design, data collection, data analysis and interpretation, and manuscript preparation. BK assisted with data interpretation and manuscript preparation. PL assisted with experimental design and manuscript preparation. DP assisted with experimental design, data analysis and interpretation, and manuscript preparation. All authors contributed to the article and approved the submitted version.

## Conflict of Interest

Authors RB and PL were employed by company Red Bull North America.

The remaining authors declare that the research was conducted in the absence of any commercial or financial relationships that could be construed as a potential conflict of interest.

## Publisher’s Note

All claims expressed in this article are solely those of the authors and do not necessarily represent those of their affiliated organizations, or those of the publisher, the editors and the reviewers. Any product that may be evaluated in this article, or claim that may be made by its manufacturer, is not guaranteed or endorsed by the publisher.

## Footnotes

^1^ http://fmri.wfubmc.edu/software/pickatlas

## Supplementary Material

The Supplementary Material for this article can be found online at: https://www.frontiersin.org/articles/10.3389/fpsyg. 2022.800275/full#supplementary-material
