# Supplementary material for: Comparison of Hemodynamic Brain Responses Between Big Wave Surfers and Non-big Wave Surfers During Affective Image Presentation
Source: Front Psychol. 2022 Jun 16;13:800275. doi: 10.3389/fpsyg.2022.800275 (PMC9245544; doi:10.3389/fpsyg.2022.800275)
Supplement: Supplementary file 1 [file Table_1.DOCX]

**Appendix: IAPS images viewed during the fMRI scan**

| IAPS image number | | | |
| --- | --- | --- | --- |
| HAN | HAP | LAN | LAP |
| 1120 | 1650 | 2039 | 1419 |
| 1201 | 8158 | 2101 | 1604 |
| 1525 | 2216 | 2104 | 1610 |
| 1930 | 4597 | 2206 | 1620 |
| 6230 | 4599 | 2210 | 1670 |
| 3005.1 | 4626 | 2215 | 1812 |
| 3015 | 4653 | 2271 | 1910 |
| 3019 | 4677 | 2272 | 2000 |
| 9901 | 4689 | 2279 | 2035 |
| 3102 | 5260 | 2280 | 1450 |
| 3170 | 5450 | 2383 | 2320 |
| 3266 | 5460 | 2440 | 2304 |
| 3500 | 5470 | 2441 | 2360 |
| 3530 | 5621 | 2491 | 2370 |
| 3350 | 5629 | 2722 | 2384 |
| 6315 | 5833 | 5120 | 2388 |
| 6300 | 7270 | 5130 | 2501 |
| 6415 | 7405 | 7016 | 2598 |
| 6560 | 7451 | 7025 | 5010 |
| 6563 | 7502 | 7030 | 5000 |
| 6570 | 7650 | 7031 | 5202 |
| 6840 | 8030 | 7040 | 5410 |
| 7380 | 8080 | 7060 | 5551 |
| 8230 | 8170 | 7078 | 5725 |
| 9075 | 8180 | 7110 | 5760 |
| 9163 | 8185 | 7130 | 5779 |
| 9300 | 8190 | 7150 | 5780 |
| 9325 | 8191 | 7180 | 5800 |
| 2717 | 8200 | 7186 | 5875 |
| 9405 | 8210 | 7224 | 5891 |
| 9412 | 8300 | 7234 | 7039 |
| 9413 | 8340 | 7287 | 7165 |
| 9560 | 8370 | 7595 | 7192 |
| 9600 | 8380 | 7700 | 7325 |
| 9630 | 8470 | 7705 | 7340 |
| 9635.1 | 8490 | 9001 | 7507 |
| 9800 | 8492 | 9210 | 7509 |
| 9810 | 8499 | 9260 | 7545 |
| 9910 | 8501 | 9360 | 7900 |
| 9940 | 8502 | 9700 | 8311 |
