# Supplementary material for: Comparison of Hemodynamic Brain Responses Between Big Wave Surfers and Non-big Wave Surfers During Affective Image Presentation
Source: Front Psychol. 2022 Jun 16;13:800275. doi: 10.3389/fpsyg.2022.800275 (PMC9245544; doi:10.3389/fpsyg.2022.800275)
Supplement: Supplementary file 2 [file Table_2.docx]

**Supplementary Table 2**: Regions showing activations of combined BWS and CON participant groups.

| **Volume** | **Peak Voxel** | **MNI Coordinates (mm)** | | | **Side** | **Brain Region** |
| --- | --- | --- | --- | --- | --- | --- |
| **(mm^3^)** | **(Z)** | **x** | **y** | **z** |  |  |
| *HAN > FIX* | | | | | | |
| 5,778 | 4.75 | -54 | -28 | -2 | L | Superior Temporal gyrus |
| 5,184 | 4.62 | -18 | 8 | 4 | L | Caudate; putamen; pallidum |
| 2,322 | 4.15 | -42 | -58 | 43 | L | Angular gyrus |
| 5,373 | 4.13 | -12 | 20 | 49 | L | Supplementary motor area |
| 2,646 | 4.05 | 21 | 23 | -2 | R | Caudate; putamen; pallidum |
| 3,942 | 4.04 | 36 | 14 | 40 | R | Frontal eye fields |
| *FIX > HAN* | | | | | | |
| 3,402 | 4.52 | -33 | -58 | -17 | L | Fusiform |
| 4,671 | 4.28 | 36 | -58 | -20 | R | Fusiform |
| 2,403 | 3.65 | 30 | -70 | 22 | R | Occipital gyrus |
| *LAN > FIX* | | | | | | |
| 1,971 | 3.82 | -54 | -19 | 4 | L | Superior Temporal gyrus |
| *FIX > HAN* | | | | | | |
| 3,699 | 4.09 | -36 | -58 | -17 | L | Fusiform |
| 5,778 | 3.84 | 24 | -70 | -17 | R | Fusiform |
| *HAN > LAN* | | | | | | |
| 1,539 | 4.07 | -18 | 11 | -2 | L | Caudate; putamen; pallidum |
| *FIX > HAN* | | | | | | |
| None |  |  |  |  |  |  |

Voxel p < 0.001 and cluster-level p < 0.05 whole-brain corrected for family-wise error of multiple comparisons. R: right; L: left.

When examining the mean BOLD signal across all participants, as detailed in Supplementary Table 1, presentation of positive-valence images resulted in minimal brain signal changes, while presentation of negative-valence images resulted in significant increases and decreases in signal intensity in localized regions of the brain at voxel p < 0.001 uncorrected and cluster-level p < 0.05, FWE corrected (Supplementary Figure 1). Of note, significant increases in activation were observed in structures in the striatum, frontal motor areas, and the temporal and angular gyri during negative image presentation. Similarly significant reductions in signal intensity were observed in the fusiform gyrus during HAN image presentation.
