# Supplementary figures and images for: Comparison of Hemodynamic Brain Responses Between Big Wave Surfers and Non-big Wave Surfers During Affective Image Presentation
Source: Front Psychol. 2022 Jun 16;13:800275. doi: 10.3389/fpsyg.2022.800275 (PMC9245544; doi:10.3389/fpsyg.2022.800275)

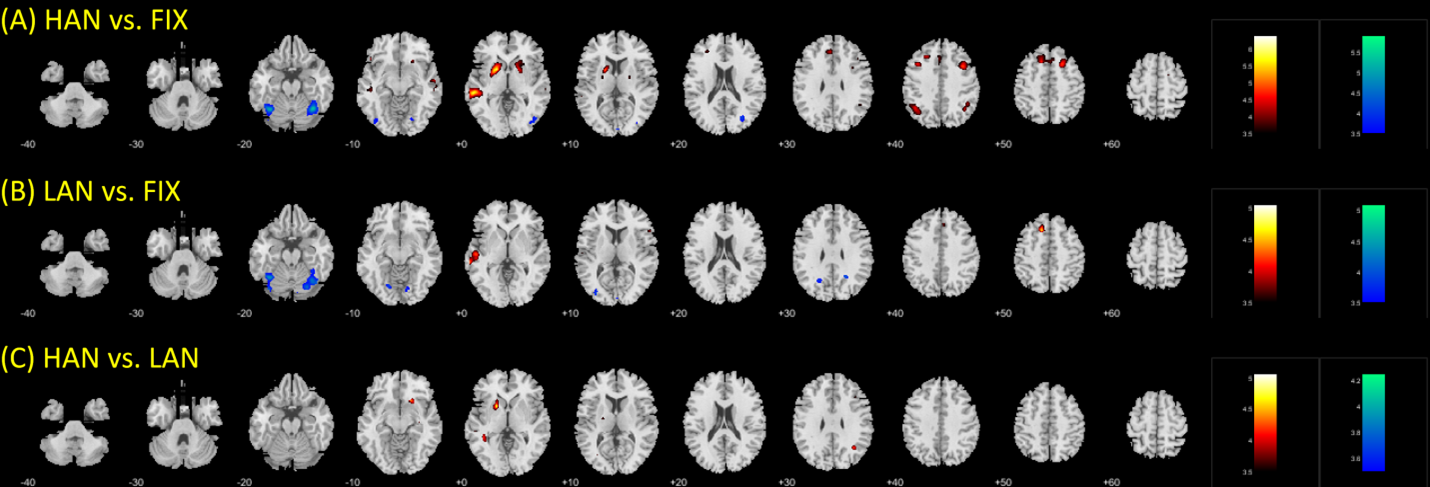

Supplement: Supplementary file 3 [file Image_1.TIF]
